# Aberrant epidermal differentiation and disrupted ΔNp63/Notch regulatory axis in Ets1 transgenic mice

**DOI:** 10.1242/bio.20135397

**Published:** 2013-10-23

**Authors:** Shu Shien Chin, Rose-Anne Romano, Priyadharsini Nagarajan, Satrajit Sinha, Lee Ann Garrett-Sinha

**Affiliations:** Department of Biochemistry, State University of New York at Buffalo, Buffalo, NY 14203, USA

**Keywords:** Transcription, Differentiation, Epidermis, Notch, Transgenic mice

## Abstract

The transcription factor Ets1 is expressed at low levels in epidermal keratinocytes under physiological conditions, but is over-expressed in cutaneous squamous cell carcinoma (SCC). We previously showed that over-expression of Ets1 in differentiated keratinocytes of the skin leads to significant pro-tumorigenic alterations. Here, we further extend these studies by testing the effects of over-expressing Ets1 in the proliferative basal keratinocytes of the skin, which includes the putative epidermal stem cells. We show that induction of the Ets1 transgene in the basal layer of skin during embryogenesis results in epidermal hyperplasia and impaired differentiation accompanied by attenuated expression of spinous and granular layer markers. A similar hyper-proliferative skin phenotype was observed when the transgene was induced in the basal layer of the skin of adult mice leading to hair loss and open sores. The Ets1-mediated phenotype is accompanied by a variety of changes in gene expression including alterations in Notch signaling, a crucial mediator of normal skin differentiation. Finally, we show that Ets1 disrupts Notch signaling in part via its ability to upregulate ΔNp63, an established transcriptional repressor of several of the Notch receptors. Given the established tumor suppressive role for Notch signaling in skin tumorigenesis, the demonstrated ability of Ets1 to interfere with this signaling pathway may be important in mediating its pro-tumorigenic activities.

## Introduction

The epidermal terminal differentiation program is orchestrated in a precise manner by distinctive sets of transcription factors and signal transduction pathways. One signaling pathway known to participate in keratinocyte differentiation is the Notch pathway, which has two distinct and important roles. First, it downregulates the basal cell phenotype and promotes differentiation by upregulating expression of differentiation markers and blocking cell cycle progression (Rangarajan et al., 2001; Moriyama et al., 2008; Restivo et al., 2011). Second, Notch prevents premature differentiation of spinous keratinocytes into granular layer keratinocytes, an activity dependent on the Notch target Hes1 (Moriyama et al., 2008). In Notch1 deficient mice, the skin is hyper-proliferative and it aberrantly expresses differentiation markers (Rangarajan et al., 2001), while in Notch1/Notch2 deficient skin, the spinous layer is largely absent (Moriyama et al., 2008). Similarly, in the absence of Hes1, spinous layer formation is impaired (Moriyama et al., 2008). Conversely, enhanced Notch signaling in the basal layer of the epidermis promotes premature differentiation into spinous keratinocytes (Blanpain et al., 2006). Notch signaling appears to function in part by driving the expression of the transcription factor Irf6 (Restivo et al., 2011), which is required for proper keratinocyte differentiation (Ingraham et al., 2006; Richardson et al., 2006).

As a counterbalance to Notch signaling, the transcription factor p63 prevents premature differentiation of keratinocytes and maintains the basal cell phenotype. This effect of p63 is largely mediated by ΔNp63, the predominant isoform expressed in the skin (Romano et al., 2009; Romano et al., 2012). Loss of all p63 isoforms or specific loss of the ΔNp63 isoform leads to severe impairments in epidermal morphogenesis and premature differentiation and is accompanied by reduced Notch1 expression in stratified squamous epithelial tissues (Mills et al., 1999; Yang et al., 1999; Laurikkala et al., 2006; Romano et al., 2012). However the effect of p63 on Notch1 expression is complex and may be dependent on specific tissues or developmental stages, since other studies have shown that ΔNp63 can directly repress expression of Notch1 and Hes1 in epidermal keratinocytes (Nguyen et al., 2006; Okuyama et al., 2007; Yugawa et al., 2010). ΔNp63 also appears to repress expression of Notch2 and Notch3 in skin (Romano et al., 2012). Finally, Notch signaling feeds back to inhibit p63 expression, thereby generating a negative regulatory loop (Nguyen et al., 2006; Okuyama et al., 2007).

The normal pattern of skin differentiation is commonly disrupted in squamous cell carcinomas (SCC). Accompanying this impaired differentiation is the frequent downregulation and/or impaired function of *Notch* and *Irf6* genes and the amplification/upregulation of p63 (Wrone et al., 2004; DeYoung et al., 2006; Agrawal et al., 2011; Botti et al., 2011; Stransky et al., 2011). The oncogene Ets1 represents another transcription factor frequently over-expressed in SCC (Pande et al., 1999; Saeki et al., 2000; Keehn et al., 2004). Ets1 is expressed at low levels under normal homeostatic conditions in the embryonic or adult epidermis, but over-expressed in SCC, particularly those that are poorly differentiated (Keehn et al., 2004). In order to understand the effects of Ets1 on the skin differentiation program and how that might contribute to its oncogenic effects, we have developed an inducible bi-transgenic (BT) mouse model in which we can mimic the effects of Ets1 over-expression. Using this inducible BT system, we have previously shown that Ets1 expression in suprabasal keratinocytes leads to dysplastic phenotypes and induction of pro-inflammatory and pro-tumorigenic genes (Nagarajan et al., 2009; Nagarajan et al., 2010). In the current study, we demonstrate that induction of Ets1 in the basal proliferative layer of the skin impairs Notch signaling at least in part through the upregulation of ΔNp63 expression.

## Results

### Ets1 over-expression during embryonic development leads to an eye-open-at-birth phenotype and perinatal lethality

**Fig. 1.**
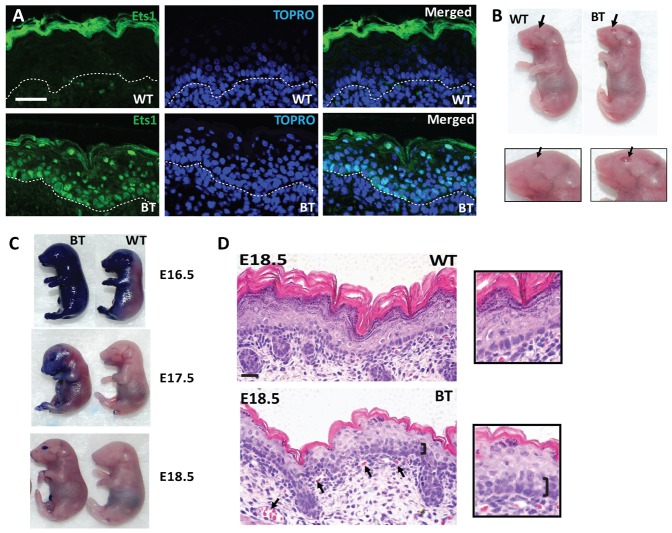
Effects of over-expression of Ets1 in the basal layer of the skin. (A) Immunofluorescent staining of E18.5 wild-type and K5-Ets1 BT skin for Ets1 (green staining). Sections were counterstained with TOPRO-3 to mark the nuclei (blue). Scale bars are 37.5 µm. (B) Embryonic E18.5 K5-Ets1 BT mice in which the Ets1 transgene was induced prenatally exhibit an eye-open-at-birth phenotype (arrows point to closed or open eyes in the wild-type and K5-Ets1 BT pups), but grossly normal skin. (C) K5-Ets1 BT embryos exhibit delayed skin barrier acquisition at E16.5 and E17.5. However, at E18.5, both wild-type and BT embryos show similar skin barrier function (note that the open-eye phenotype of BT embryos results in a blue-stained eye). (D) Hematoxylin and eosin (H&E) staining of E18.5 embryonic wild-type and BT skin demonstrates a second layer of basal-like epithelial cells (black bracket), impaired differentiation of suprabasal keratinocytes and increased angiogenesis (black arrows). Scale bars are 37.5 µm. Inset shows a higher magnification view of the skin.

We examined the effects of expressing Ets1 in the basal layer of the skin by crossing an Ets1 responder transgenic line (Nagarajan et al., 2009; Nagarajan et al., 2010) to a driver transgenic line that expresses the tetracycline transactivator protein (tTA) under the control of the K5 promoter (Diamond et al., 2000). The resulting K5-Ets1 bi-transgenic (BT) offspring can be induced to express Ets1 in the undifferentiated, proliferating layers of the skin epidermis and other stratified squamous epithelia during embryonic development by withholding doxycycline (Dox) from the pregnant dams. Immunofluorescence staining for Ets1 in late-stage embryos revealed that transgenic Ets1 expression was robust in the basal layer of the BT skin as expected, whereas endogenous Ets1 in wild-type controls was undetectable under these conditions (Fig. 1A). A high level of Ets1 expression in BT skin was confirmed by Western blotting with skin lysates from BT animals (not shown). Interestingly, transgenic Ets1 expression was not restricted to only the basal cells, but also extended to some degree in the suprabasal layers. This, we posit, could reflect the retention of stable, transgenic Ets1 protein after transition from the basal to differentiated layers or perhaps suprabasal expression of the transgenic protein due to abnormal activation of K5 promoter. An alternate possibility might be the induction of endogenous Ets1 in response to transgene-induced skin alterations.

None of the newborn BT animals survived for longer than 24 hours after birth. Newborn BT mice had skin that appeared grossly normal, but they were characterized by an eye-open-at-birth phenotype (Fig. 1B). This phenotype made them easily distinguishable from their littermate controls. Dye exclusion assays demonstrated that the acquisition of skin barrier function of K5-Ets1 BT embryos was somewhat delayed at embryonic day (E) 16.5 and 17.5, but caught up in E18.5 embryos (Fig. 1C). This suggests that the post-natal lethality is not due to skin barrier defects, but may instead be due to alterations in other K5+ epithelia such as the oral or esophageal epithelium.

### Ets1 over-expression drives an expansion of a basal-like keratinocyte population

To identify potential Ets1-driven alterations in skin epithelium, we examined E18.5 embryos in which Ets1 was induced throughout gestation. Hematoxylin and eosin (H&E) staining of skin sections revealed multiple alterations in the normal skin differentiation program (Fig. 1D and higher magnification inset), that partially overlapped with the skin phenotype seen in mice over-expressing Ets1 in the differentiated layers of the skin (Inv-Ets1 BT mice) (Nagarajan et al., 2009). Interestingly and unexpectedly, we observed two cellular layers with basal-like morphology (cuboidal cells with darkly staining nuclei) in K5-Ets1 BT skin, suggesting that cells that had just exited the basal layer failed to adopt proper spinous characteristics (black bracket, Fig. 1D). This phenotype was not observed previously in Inv-Ets1 BT skin and hence represents a specific effect of expressing Ets1 in the basal layer of the skin. Furthermore, there was increased angiogenesis and an accumulation of immune cells in the skin of K5-Ets1 BT mice (Fig. 1D). Since these infiltrating immune cells were present in embryonic skin, which was harvested from the sterile uterine environment, the recruitment of immune cells is independent of overt infection. Immune cells found in embryonic K5-Ets1 BT skin were mainly CD11b+ and hence primarily innate immune cells such as macrophages and granulocytes (data not shown).

**Fig. 2.**
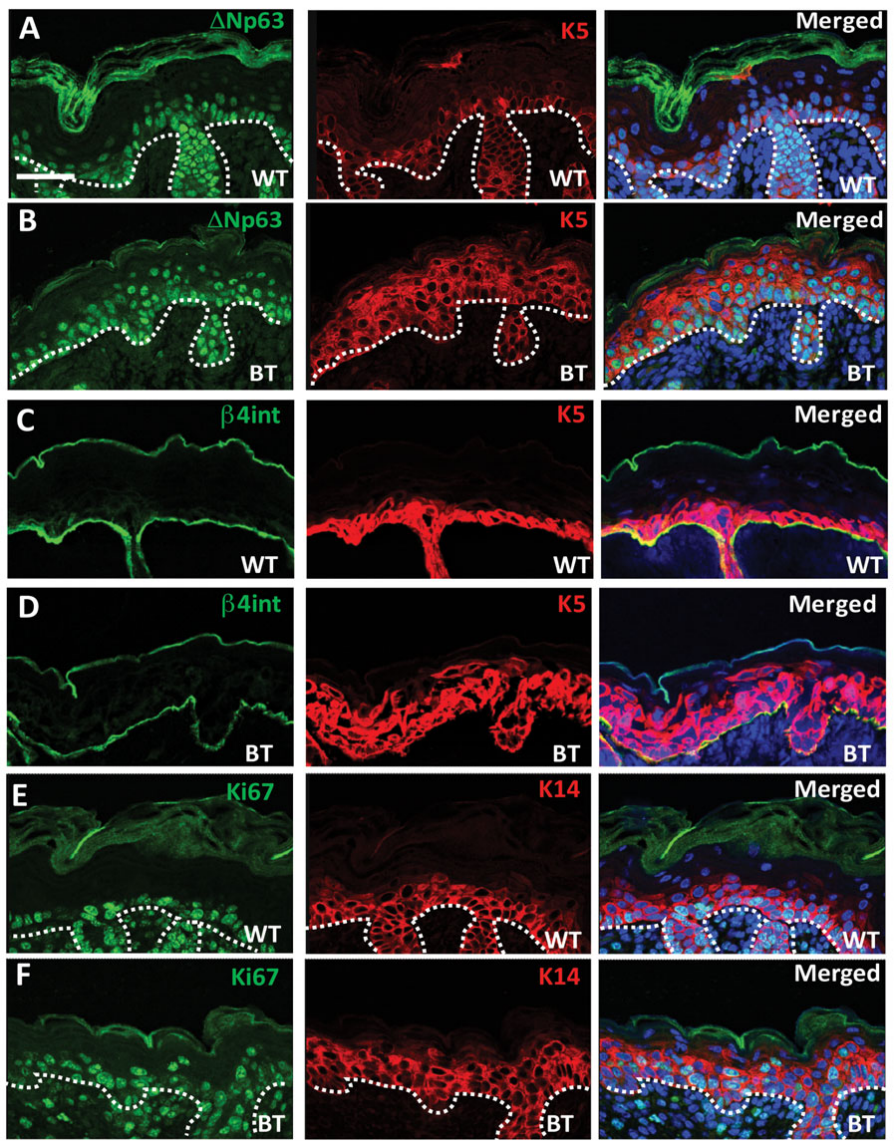
K5-Ets1 BT skin demonstrates an expansion of the basal layer. Immunofluorescent staining of E18.5 wild-type and K5-Ets1 BT skin for ΔNp63 (A–B), β4-integrin (C–D) and Ki67 (E–F) (green staining for all). Sections were also co-stained with antibodies to K5 or K14 (red) and with TOPRO-3 to mark the nuclei (blue). Scale bars are 37.5 µm in all cases.

To better understand the effects of Ets1 over-expression on the proliferative basal keratinocytes, we stained skin sections for a series of markers. Expression of keratin 5 (K5) was expanded in BT epidermis, whereas in wild-type controls, K5 staining was largely restricted to the basal layer (Fig. 2A–D). A similar staining pattern was observed for K14, the K5 partner (Fig. 2E,F). This expanded K5/K14 staining pattern is similar to what we had previously noticed in Inv-Ets1 BT skin (Nagarajan et al., 2009; Nagarajan et al., 2010). We sought to further confirm expansion of the basal layer keratinocytes by investigating the expression of other basal markers. In BT skin more keratinocytes in the basal layer and immediately suprabasal layer expressed the key basal transcription factor ΔNp63 (Fig. 2A,B). Quantification of the numbers of stained nuclei indicated that the average section of wild-type skin had 54.5±3.5 ΔNp63+ nuclei, while K5-Ets1 BT skin had 102±24.2 ΔNp63+ nuclei (*P*<0.05). In contrast, the expression of basal specific β4-integrin in K5-Ets1 BT skin was not significantly different from wild-type skin (Fig. 2C,D). The hyper-proliferative state of the epidermis was confirmed by staining for Ki67, which was expressed in both the basal and immediate suprabasal keratinocytes of BT mice, but only in basal keratinocytes of wild-type mice (Fig. 2E,F). In contrast, there was no increase in apoptosis in K5-Ets1 BT keratinocytes as determined by staining for the active form of caspase-3 (data not shown).

### Impaired terminal differentiation in K5-Ets1 BT skin

**Fig. 3.**
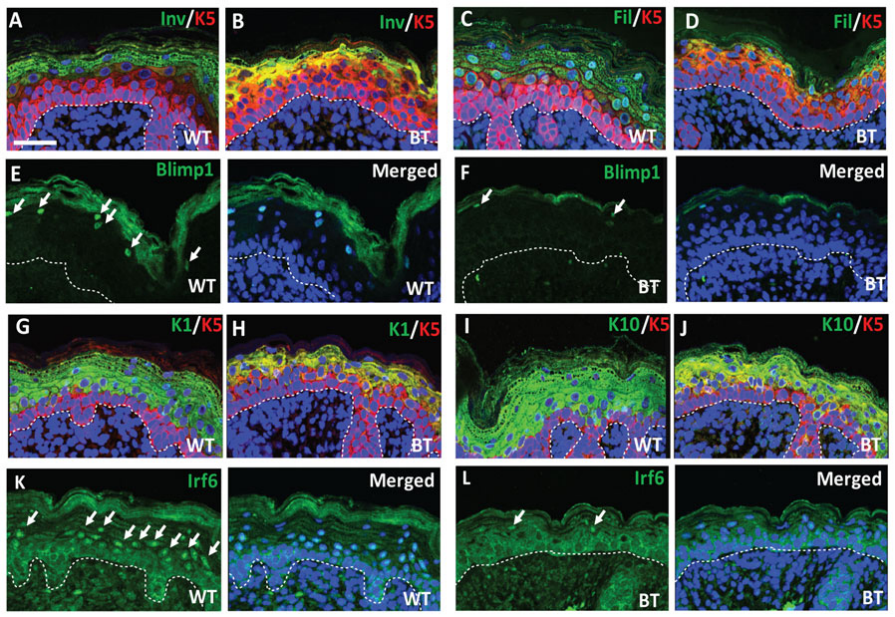
Expression of spinous layer and granular layer markers is decreased in K5-Ets1 BT skin. Immunofluorescent staining of wild-type and K5-Ets1 BT E18.5 skin using antibodies specific for spinous and granular layer markers (green). Involucrin (Inv, A–B), filaggrin (Fil, C–D), Blimp1 (E–F), K1 (G–H), K10 (I–J) and Irf6 (K–L). White arrows point to Blimp1+ or Irf6+ keratinocytes. Some panels were also co-stained with antibodies to K5 (red). All sections were stained with TOPRO-3 to mark the nuclei (blue). Scale bars in all cases are 37.5 µm.

In contrast to the seeming duplication of the basal layer, the differentiating layers of the epidermis were reduced and compacted as observed in H&E stained sections of K5-Ets1 BT embryos (Fig. 1D). These alterations were particularly pronounced for the darkly-staining granular layer of the skin and the cornified layer, whereas the spinous layer was apparently preserved. In keeping with this histological appearance, K5-Ets1 BT skin demonstrated diminished expression of granular layer markers such as involucrin, filaggrin and loricrin (Fig. 3A–D and data not shown). Furthermore, expression of the transcription factor Blimp1, marking a subset of cells in the granular layer (Magnúsdóttir et al., 2007), was also reduced in K5-Ets1 BT skin (Fig. 3E,F).

Although histologically the spinous layer of K5-Ets1 BT skin did not show any marked alterations, immunostaining for the spinous layer markers K1 and K10 showed that their expression was significantly impaired in K5-Ets1 BT embryos (Fig. 3G–J). Because of the expansion in K5 expression, most suprabasal keratinocytes co-expressed both K5 and differentiation markers such as K1, K10, involucrin and filaggrin. Expression of Irf6, a transcription factor predominantly found in spinous keratinocytes, was considerably diminished in the suprabasal layers of K5-Ets1 BT skin (Fig. 3K,L). Instead, the hyper-proliferative marker keratin K6 was abnormally expressed in the suprabasal epidermal layers of BT mice (data not shown).

### Induction of Ets1 in the basal layer of adult epidermis leads to significant dysplasia

**Fig. 4.**
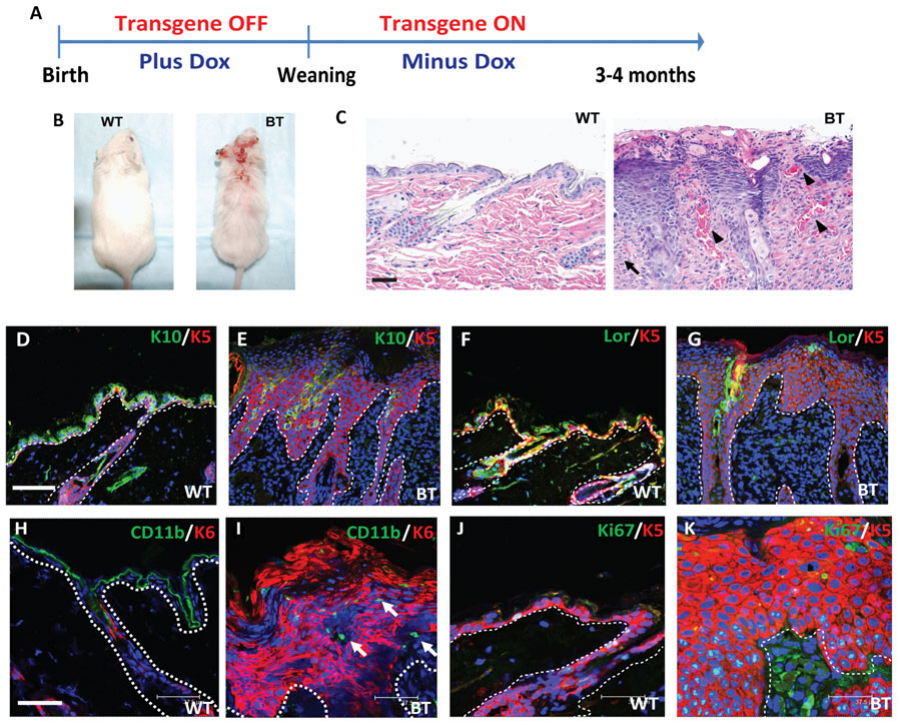
K5-Ets1 BT adult animals suffer from a dramatic skin phenotype. (A) Overview of the time course for induction of the Ets1 transgene in adult K5-Ets1 BT mice. (B) Adult BT mice induced at weaning develop non-healing sores and scabs on the skin after 3–4 months. (C) Hematoxylin and eosin (H&E) staining of adult wild-type and K5-Ets1 BT skin demonstrates hyper-proliferation, impaired differentiation, increased angiogenesis (black arrowheads) and infiltration of mononuclear cells (black arrows). Scale bars are 75 µm. (D–K) Immunofluorescent staining of wild-type and BT adult skin using antibodies specific for K10, loricrin (Lor), CD11b and Ki67 (all green). Each section was co-stained with either K5 or K6 (red) and with TOPRO-3 to mark nuclei (blue). Note overlap of green Ki67 staining with blue TOPRO-3 staining in parts J-K leads to a pale blue color in nuclei. Scale bars for D–G are 75 µm and for H–K are 37.5 µm.

K5^+^ basal layer keratinocytes include epidermal stem cells in the interfollicular epidermis and the hair follicle bulge region, which are thought to be the targets of pro-carcinogenic changes that induce tumor development (Colmont et al., 2012). Hence, we expected that expression of Ets1 in the basal layer might result in a more striking, pro-tumorigenic phenotype as compared to its expression in differentiated layers. To test this Ets1 was suppressed during embryonic and early post-natal development by administration of Dox, and then induced at weaning by withdrawing Dox supplementation (Fig. 4A). In contrast to the dramatic skin phenotypes that developed in adult Inv-Ets1 BT mice after only 3–4 weeks of induction when subjected to a similar treatment (Nagarajan et al., 2009), milder skin phenotypes developed in adult K5-Ets1 BT mice after 3–4 months, which included progressive hair loss and open sores that failed to heal (Fig. 4B). Skin lesions were frequently found on the head, neck and back, with other areas being less frequently affected. Histological analysis of lesions revealed that the epidermis was hyper-proliferative with altered differentiation (Fig. 4C). Similar results were obtained with skin sections in other areas of the body, although to a lesser degree of severity (data not shown). Immunostaining for K1, K10, loricrin, involucrin and filaggrin (Fig. 4D–G and data not shown) demonstrated that all of these markers exhibited a discontinuous and patchy expression in affected skin. Furthermore, K6 and the Ki67 antigen were aberrantly expressed in the BT skin in agreement with the hyper-proliferative state (Fig. 4H–K).

### Notch signaling is impaired in the BT epidermis

Embryonic K5-Ets1 BT mice exhibit an expanded basal layer and impaired expression of spinous layer markers, phenotypes not found in embryonic Inv-Ets1 BT mice (Nagarajan et al., 2010). To probe this mechanistically, we performed microarray analysis comparing wild-type and K5-Ets1 BT E18.5 embryonic skin. We noted many of the same changes that we have previously detected in Inv-Ets1 BT skin (Nagarajan et al., 2010), including impaired expression of cornified envelope genes and increased expression of matrix metalloprotease genes, EGF ligands, cytokines and chemokines (data not shown). We also detected alteration in components of the Notch signaling pathway in K5-Ets1 BT skin, which was not significantly affected in Inv-Ets1 BT skin. Previous studies have revealed that Notch signaling is essential for the induction of spinous fate and repression of basal fate during epidermal differentiation (Rangarajan et al., 2001; Blanpain et al., 2006; Moriyama et al., 2008). Hence, we focused further studies on the Notch pathway to understand how changes in Notch signaling might lead to the impairment in basal to spinous differentiation that is specific to K5-Ets1 BT mice.

**Fig. 5.**
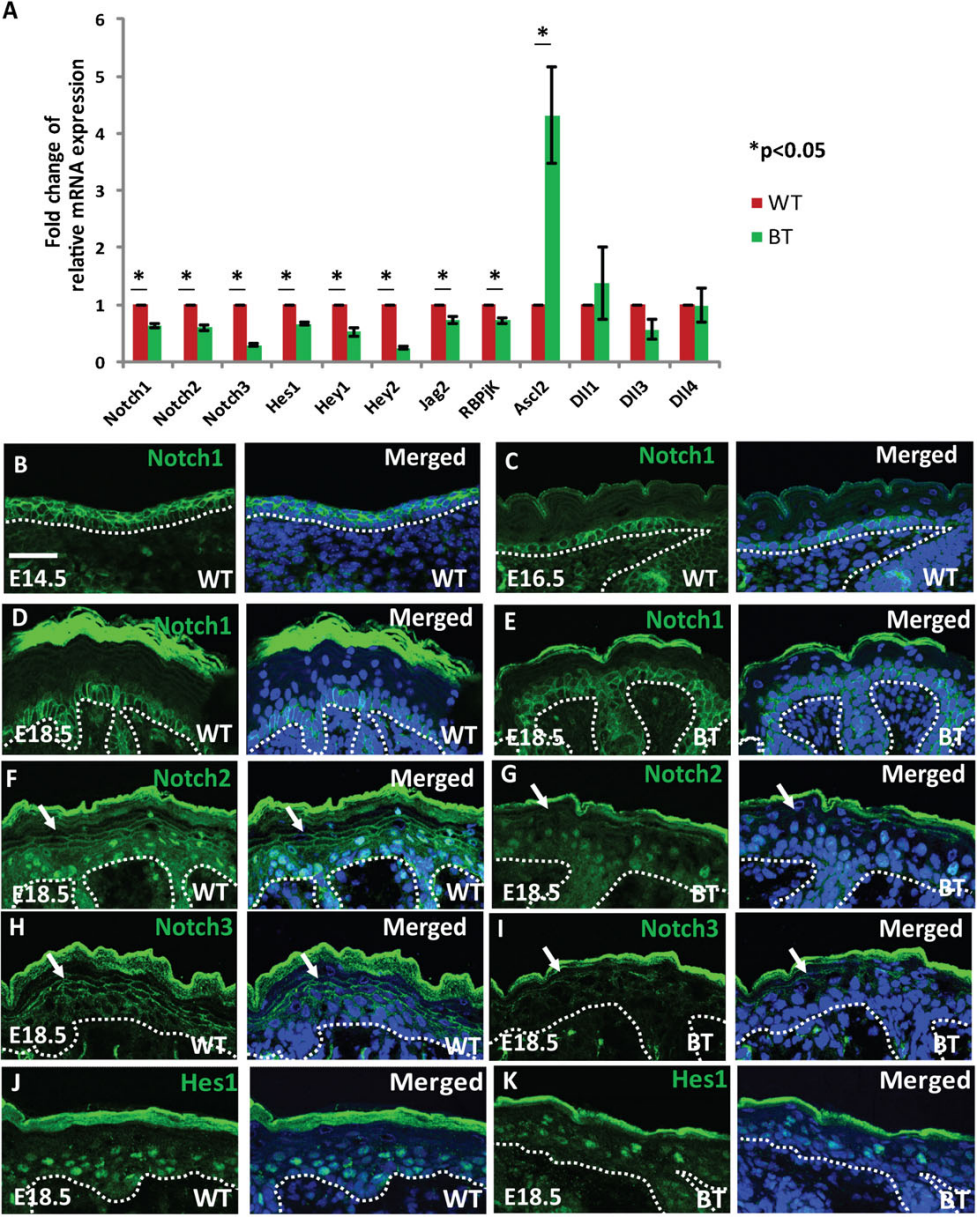
Impaired Notch signaling in K5-Ets1 BT skin. (A) Quantitative real-time PCR to measure mRNA levels of genes involved in the Notch signaling pathway. Data are represented as mean ± SEM. *P<0.05, Student's *t*-test. (B–E) Immunofluorescent staining for Notch1 in the skin of E14.5, E16.5 and E18.5 wild-type and E18.5 K5-Ets1 BT embryos (all green). (F–K) Immunofluorescent staining for Notch2, Notch3 and Hes1 in the skin of E18.5 wild-type and K5-Ets1 BT embryos (all green). White arrows point to membrane-associated staining of Notch2 and Notch3. Each section was co-stained with TOPRO-3 to mark nuclei (blue). Scale bars in all cases are 37.5 µm.

We first examined the expression levels of various Notch signaling pathway components using quantitative RT-PCR, which demonstrated a reduction in the expression of Notch1, Notch2 and Notch3 receptors as well as the downstream effectors *Hes1*, *Hey1*, *Hey2*, *Jag2* and *Rbpjk* (Fig. 5A). By immunostaining, we examined expression of Notch1, Notch2, Notch3 and Hes1. Unexpectedly, using two different monoclonal antibodies, we found Notch1 to be primarily confined to the basal layer of the skin of E18.5 embryos (Fig. 5B,C), contrary to its reported suprabasal expression (Rangarajan et al., 2001). Further analysis suggested that the Notch1 expression pattern undergoes dynamic changes during embryonic development. While at E14.5, Notch1 staining is entirely suprabasal (Fig. 5B), by E16.5 and E18.5 the staining switches to the basal layer (Fig. 5C,D). In E18.5 BT epidermis, overall Notch1 staining was not reduced, but rather expanded to the duplicated basal layer (Fig. 5D,E). However, the Notch1 staining appeared to be somewhat more patchy and irregular in K5-Ets1 BT skin than in wild-type skin. Notch2 and Notch3 receptors were continuously expressed along the cell membrane of suprabasal keratinocytes in wild-type skin, whereas in the K5-Ets1 BT skin Notch2 and Notch3 staining was significantly reduced (Fig. 5F–I). Notch2 staining was also observed in the nucleus of both wild-type and K5-Ets1 BT keratinocytes, indicative of activation of Notch2 signaling.

As described above, the expression of the Notch target genes Hes1, Hey1 and Hey2 was significantly diminished in K5-Ets1 BT skin (Fig. 5A) and we confirmed downregulation of Hes1 by immunostaining (Fig. 5J,K). Notch signaling also induces expression of the achaete-scute complex homolog 2 (*Ascl2*) gene. Ascl2 and Hes1 have counterbalancing roles in skin homeostasis. Ascl2 drives terminal differentiation of cells that have exited the basal layer into a granular layer fate, while Hes1 represses expression of Ascl2, thereby allowing the formation of spinous cells to occur (Moriyama et al., 2008). In keeping with decreased Hes1 expression detected in K5-Ets1 BT skin, the expression of the *Ascl2* gene was upregulated (Fig. 5A).

### Ets1 triggers increased expression of the Notch repressor ΔNp63

**Fig. 6.**
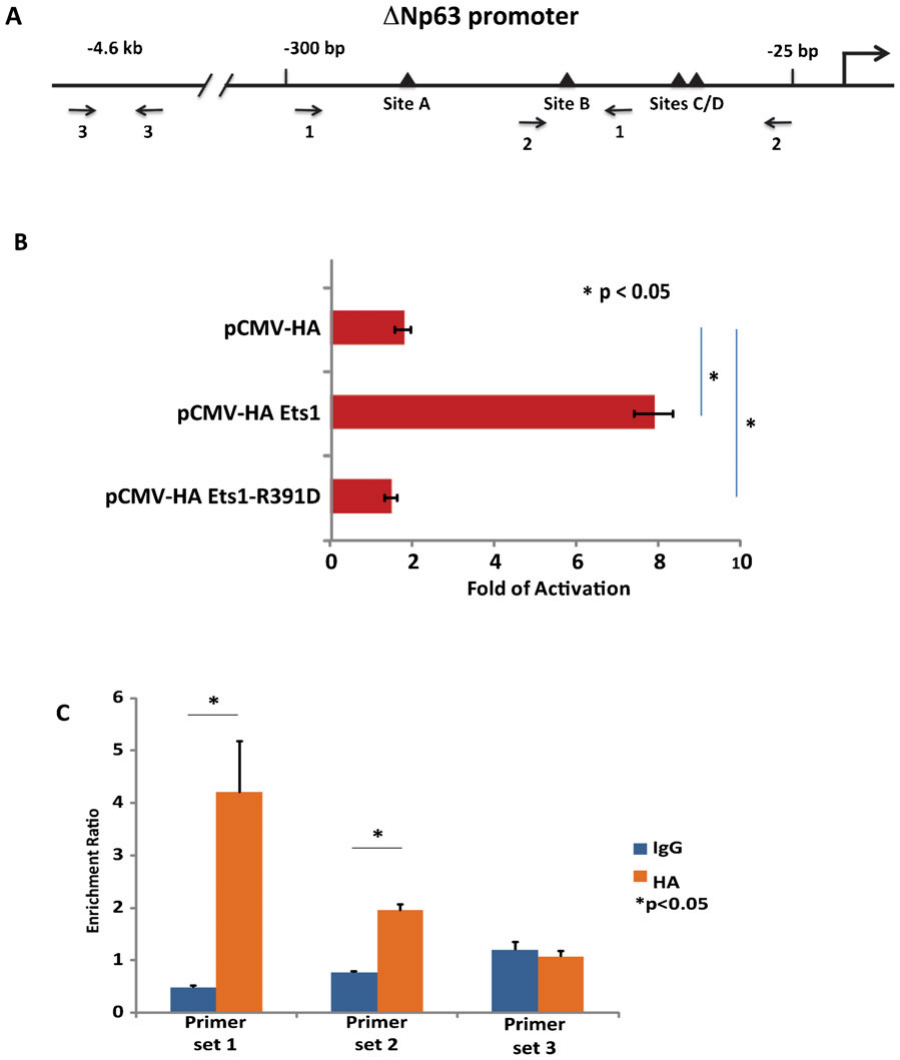
Ets1 regulates expression of ΔNp63. (A) Diagram of the ΔNp63 proximal promoter with potential Ets1 binding sites indicated by black triangles. Arrows below indicate three primer sets used in the ChIP assay. (B) Normalized luciferase activity of the ΔNp63 promoter co-transfected with empty vector (pCMV-HA), a plasmid encoding wild-type Ets1 (pCMV-HA-Ets1) or a DNA-binding mutant of Ets1 (pCMV-HA-Ets1-R391D). (C) ChIP assay using chromatin derived from HA-Ets1 expressing keratinocytes and primer sets 1 (surrounds sites A and B), 2 (surrounds sites B, C and D) and 3 (lacks potential Ets binding sites).

While Notch signaling promotes differentiation and inhibits proliferation of keratinocytes, the transcription factor ΔNp63 plays the opposite roles. ΔNp63 can directly repress expression of Notch genes in skin (Nguyen et al., 2006; Okuyama et al., 2007; Yugawa et al., 2010; Romano et al., 2012). As described above, expression of the ΔNp63 is significantly elevated in basal and suprabasal layers of K5-Ets1 BT skin (Fig. 2A,B). We hypothesized Ets1 might act to upregulate expression of ΔNp63, thus leading to impaired Notch activity. Indeed, the ΔNp63 proximal promoter harbors several potential Ets1 binding sites as defined by the presence of the core Ets binding motif GGA^A^/_T_ (Fig. 6A). We reasoned that one or more of these potential Ets1 binding elements might contribute to ΔNp63 induction in the K5-Ets1 BT mice. To test the ability of Ets1 to transactivate the ΔNp63 promoter, an expression plasmid encoding Ets1 was transfected along with a ΔNp63 promoter driven luciferase reporter construct into mouse keratinocytes. ΔNp63 promoter activity was significantly upregulated by Ets1, but not by a DNA-binding deficient Ets1 mutant (R391D) (Fig. 6B).

To further validate that Ets1 can bind to ΔNp63 promoter in a genomic context we performed a chromatin immunoprecipitation (ChIP) assay using chromatin derived from cultured mouse keratinocytes infected with retrovirus that expresses an HA-tagged version of Ets1. We chose this approach because the level of Ets1 in cultured keratinocytes is low and it was not feasible to immunoprecipitate the endogenous protein under these conditions. As shown in Fig. 6C, HA-tagged Ets1 was recruited to the ΔNp63 proximal promoter region that contains putative Ets1-binding sites (primer sets 1 and 2), but not a distal upstream region (primer set 3), strongly suggesting that the ΔNp63 promoter is a direct target of Ets1.

## Discussion

### Oncogenic effects of Ets1

Ets1 was originally cloned from an oncogenic retrovirus and has been shown to be over-expressed in many human tumors, where high levels of expression are correlated with tumor aggression and invasion (Dittmer, 2003; Lincoln and Bove, 2005). Previously we have examined the effects of expressing the oncogenic transcription factor Ets1 in the differentiated layers of the skin epidermis, where it drives a number of pro-tumorigenic changes including hyper-proliferation and impaired differentiation coupled with enhanced expression of matrix metalloproteases and inflammatory mediators (Nagarajan et al., 2009; Nagarajan et al., 2010). A prevalent hypothesis argues that tumor-promoting events, such as upregulated expression of oncogenes, take place in tissue stem cells (Visvader, 2011; Colmont et al., 2012). However, there are also data indicating that cancers may arise from more differentiated cells that undergo de-differentiation (Torres-Montaner, 2011). In our current study, we wished to determine whether expression of Ets1 in K5^+^ basal layer keratinocytes would promote more significant pro-tumorigenic changes than those that occur in when Ets1 is expressed in more differentiated cells of the epidermis.

Induction of Ets1 in the basal layer of the epidermis in K5-Ets1 BT mice did lead to significant alterations in the skin. However the phenotypes that developed were in general milder than those found when Ets1 was induced in the differentiated layers of the skin in Inv-Ets1 BT mice. Although it is possible that these differences could be explained by the overall level or extent of Ets1 expression driven by the K5 transgenic driver line versus the involucrin transgenic driver line, we think this is unlikely as Western blotting showed similar levels of Ets1 over-expression in both K5-Ets1 and Inv-Ets1 BT skin (data not shown).

Inv-Ets1 BT mice, but not K5-Ets1 BT mice, exhibit a significant skin barrier defect in late embryogenesis just prior to birth. Inv-Ets1 BT mice also show a more dramatic induction of some pro-tumorigenic genes, such as matrix metalloproteases (*Mmp1a*, *Mmp1b*, *Mmp8* and *Mmp13*), chemokines (*Ccl2* and *Cxcl5*) and EGF ligands (*Tgfa* and *Hbegf*), in microarray analyses than do K5-Ets1 BT mice. These observations suggest that Ets1 expression in differentiated keratinocytes might have a stronger oncogenic effect than Ets1 expression in undifferentiated keratinocytes and epidermal stem cells. In this fashion Ets1 may share similarities with the oncogenic transcription factor c-myc, which has previously been shown to have a more pro-oncogenic activity when expressed in the supra-basal differentiated layers of the epidermis than in the basal undifferentiated layer (Pelengaris et al., 1999; Arnold and Watt, 2001).

### Alterations to skin differentiation in K5-Ets1 BT mice

The skin of K5-Ets1 BT mice and the skin of Inv-Ets1 BT mice demonstrate overlapping phenotypes in that expression of cornified envelope genes is impaired, while expression of the basal markers is expanded in both genotypes of mice ((Nagarajan et al., 2009; Nagarajan et al., 2010) and this report). However, significant differences are found between these strains as well. Expression of spinous layer markers is largely unchanged in Inv-Ets1 BT skin (Nagarajan et al., 2009; Nagarajan et al., 2010), but is impaired in K5-Ets1 BT skin. Furthermore, K5-Ets1 BT skin, but not Inv-Ets1 skin, showed an apparent duplication of the basal layer of the epidermis. The cells comprising this duplicated basal layer appear to be in an arrested state of differentiation in which they retain morphological characteristics of basal epidermal cells and the expression of some basal-specific markers such as K5, K14 and ΔNp63, but fail to express β4-integrin.

In the Inv-Ets1 BT model, the development of the granular and cornified layers of the skin is more significantly impaired than in K5-Ets1 BT mice as assessed by microarray, qPCR, Western blotting and immunostaining. This is likely due to higher levels of Ets1 in the differentiated granular layers of Inv-Ets1 BT mice than K5-Ets1 BT mice and might in part explain why Inv-Ets1 BT embryos exhibit a skin barrier defect at E18.5 (Nagarajan et al., 2010), but K5-Ets1 BT embryos do not. The changes to stratified squamous epithelium caused by Ets1 induction in K5+ basal cells leads to perinatal lethality, despite the fact that the skin barrier function seems largely intact by late gestation based on dye exclusion tests. This suggests that the post-natal lethality is not due to skin barrier defects, but may instead be due to alterations in other K5+ epithelia such as the oral or esophageal epithelium. Overall it would appear that expression of Ets1 in the basal layer interferes with differentiation of basal keratinocytes to spinous keratinocytes, while expression of Ets1 in the granular layer impairs formation of the granular layer.

### Molecular mechanisms underlying the phenotype

Expression of Ets1 in the basal layer of the skin of K5-Ets1 BT mice triggers alterations in the Notch signaling pathways, which are not significantly altered in Inv-Ets1 mice. Notch signaling is known to promote differentiation of keratinocytes from basal to suprabasal fates and to maintain the spinous layer (Rangarajan et al., 2001; Blanpain et al., 2006; Moriyama et al., 2008; Restivo et al., 2011). In K5-Ets1 BT skin, the level of expression of the Notch2 and Notch3 is significantly downregulated at both the protein and mRNA levels. Notch1 also showed downregulation at the mRNA level, but the protein levels appeared fairly normal in immunostaining. In keeping with the downregulation of Notch receptors, downstream effectors of the Notch pathways, including Hes1, Hey1, Hey2 and Irf6 were all downregulated as well. In contrast, expression of the *Ascl2* gene was significantly upregulated in K5-Ets1 BT skin. Ascl2 is known to drive differentiation of keratinocytes into a granular layer phenotype and its expression is normally repressed by Hes1 to prevent premature skin differentiation (Moriyama et al., 2008). The over-expression of *Ascl2* combined with decreased Hes1 would promote premature differentiation of spinous keratinocytes in K5-Ets1 BT skin. Collectively, these molecular alterations likely contribute to the delay in differentiation of cells leaving the basal layer resulting in a failure to adopt a proper spinous morphology and to apparent duplication of the basal layer.

**Fig. 7.**
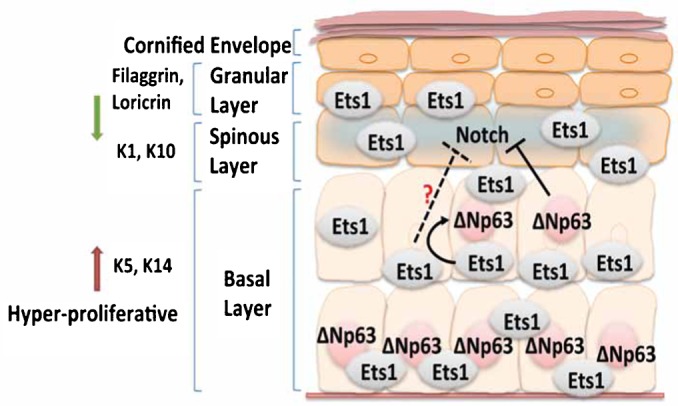
Model of Ets1 regulation of Notch signaling. When Ets1 is over-expressed it leads to upregulation of ΔNp63, which then inhibits Notch signaling and thereby prevents the transition of keratinocytes from basal to spinous cell fates. The dashed line indicates the possibility that Ets1 might also directly regulate expression of Notch genes in addition to its indirect control of Notch signaling via upregulation of ΔNp63.

Previously published data indicate that the basal cell specific transcription factor ΔNp63 may either stimulate expression of Notch receptors (Laurikkala et al., 2006) or inhibit their expression (Nguyen et al., 2006; Okuyama et al., 2007; Yugawa et al., 2010; Romano et al., 2012), depending on the tissue examined and the developmental stage. ΔNp63 can also directly repress expression of the Notch target gene Hes1 (Nguyen et al., 2006; Okuyama et al., 2007). K5-Ets1 BT epidermis shows increased ΔNp63 staining, which extends into the duplicated basal layer. This expansion of ΔNp63 expression would result in impaired Notch signaling in K5-Ets1 BT skin and delayed differentiation of spinous keratinocytes. Ets1 directly binds to the promoter region of ΔNp63 to upregulate its expression. Thus, we propose a model in which increased Ets1 expression leads to upregulation of ΔNp63, which subsequently interferes with Notch signaling and impairs keratinocyte differentiation (Fig. 7). Given that previous studies have also suggested that p63 can function as an upstream regulator of Ets1 (Candi et al., 2006), there is a strong likelihood of a transcriptional crosstalk between these two factors. It is also possible that Ets1 has a direct regulatory effect on Notch genes (shown by the dashed line in Fig. 7) – this is currently under investigation in our laboratory. In conclusion, our work is the first to demonstrate a role for the oncogene Ets1 in regulating Notch signaling to impair epidermal differentiation. Given the tumor suppressor activity of Notch signaling in SCC (Proweller et al., 2006; Lefort et al., 2007; Kolev et al., 2008; Agrawal et al., 2011; Stransky et al., 2011), Ets1's ability to block to Notch activity may be important for its pro-oncogenic effects.

## Materials and Methods

### Generation of transgenic animals

All animal experiments were performed in compliance with SUNY at Buffalo IACUC regulations. The Ets1 responder transgenic line and the K5-tTA driver transgenic mice (previously described in Diamond et al., 2000; Nagarajan et al., 2009) were crossed to generate bi-transgenic mice. Induction of the Ets1 transgene in adult mice and embryos followed previously established protocols (Nagarajan et al., 2009; Nagarajan et al., 2010).

### Immunostaining and Western blotting

Immunofluorescent staining was performed on paraffin-embedded or frozen skin sections as described (Nagarajan et al., 2010). Primary antibodies used in this study were Ets1 (Epitomics), Notch1 (Cell Signaling and Epitomics), Notch2 (Developmental Studies Hybridoma Bank), Notch3 (BioLegend), Hes1 (a gift from Dr Elaine Fuchs, Rockefeller University, New York, NY), Blimp1 (Santa Cruz) and Irf6 (R&D Systems). Antibodies to keratinocyte marker proteins have been previously described (Nagarajan et al., 2010). Counterstaining with TOPRO-3 was used to mark nuclei. Western blotting was performed as described (Romano et al., 2006; Nagarajan et al., 2010).

### Real-time qPCR

Total RNA was extracted from dorsal skin of E18.5 wild-type and BT embryos. cDNA was synthesized and qPCR was performed using SYBR green. Expression of the house-keeping gene *Gapdh* was used to normalize data. Differential gene expression was determined using the ΔΔCt method. Primer sequences are provided in Table 1.

**Table 1.**
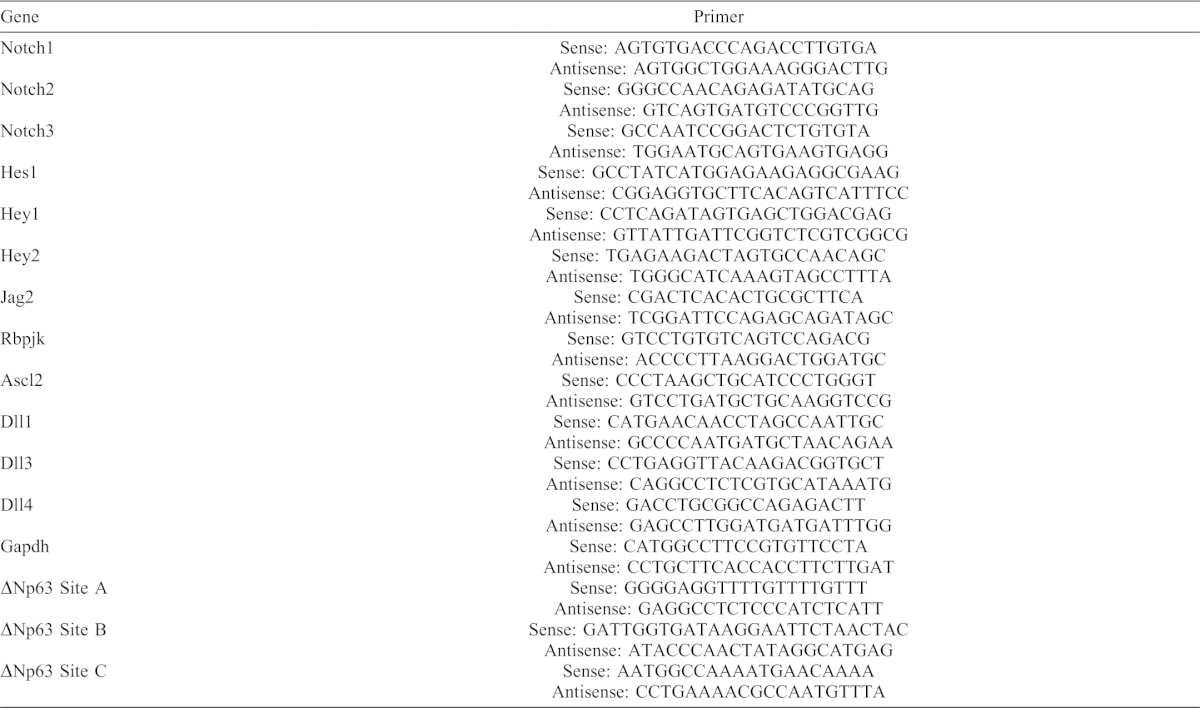
Primers. Sequences of primers used in the real-time quantitative RT-PCR and ChIP assays.

### Cell culture and transfection

The mK keratinocyte cell line and the ΔNp63 promoter firefly luciferase reporter constructs have been previously described (Romano et al., 2006). Luciferase reporter plasmids were co-transfected with an internal control plasmid pEF-RLuc, carrying a *Renilla* luciferase reporter gene and with expression plasmids carrying wild-type Ets1 (Wang et al., 2005) or mutant Ets1 (R391D) in the pCMV-HA vector. Average firefly luciferase values, normalized for Renilla luciferase, were calculated using 3 independent transfections.

### Chromatin immunoprecipitation

mK cells were infected with a retrovirus encoding HA-tagged Ets1 or a control empty virus. Chromatin was immunoprecipitated with a ChIP-validated anti-HA antibody (Abcam) using techniques previously described (Romano et al., 2012). Primer sequences used in qPCR are described in Table 1.
